# Diagnosing and treating esophageal obstruction in camels (*Camelus dromedarius*)

**DOI:** 10.14202/vetworld.2023.735-742

**Published:** 2023-04-13

**Authors:** Madeh Sadan, Sabry El-Khodery, Saleh Almatroodi, Fahd Alsobayil, El-Sayed El-Shafaey

**Affiliations:** 1 Department of Veterinary Medicine, College of Agriculture and Veterinary Medicine, Qassim University, Qassim, P.O. Box 51452, Saudi Arabia; 2 Department of Surgery, Anesthesiology, and Radiology, Faculty of Veterinary Medicine, South Valley University, Qena 83523, Egypt; 3 Department of Internal Medicine and Infectious Diseases, Faculty of Veterinary Medicine, Mansoura University, Mansoura 35516, Egypt; 4 Department of Medical Laboratories, College of Applied Medical Sciences, Qassim University, Qassim, P.O Box 51452, Saudi Arabia; 5 Department of Surgery, Anesthesiology, and Radiology, Faculty of Veterinary Medicine, Mansoura University, Mansoura 35516, Egypt

**Keywords:** animals, diagnostic imaging, esophagus, fistula, pathology

## Abstract

**Background and Aim:**

Esophageal obstruction is a common occurrence and a serious condition in camels. This study aimed to assess the effects of mineral deficiency on esophageal obstruction rates in dromedary camels and describe their clinical presentation and treatment outcomes.

**Materials and Methods:**

Twenty-eight camels were allocated to two groups. Group 1 (control) was composed of 10 sound camels. Group 2 included 18 camels with esophageal obstruction which were based on clinical and imaging evaluations. Hematobiochemical examinations in control and affected camels were compared and statistically analyzed.

**Results:**

In camels with esophageal obstruction when compared with controls, hematological analyses showed significant increases (p < 0.05) in neutrophils, lymphocytes, and monocytes, along with significantly decreased total white blood counts. Aspartate transaminase, alanine transaminase, alkaline phosphatase, creatine phosphokinase, glucose, albumin, creatinine, and blood urea nitrogen concentrations were significantly higher in affected camels when compared with controls. Furthermore, gamma-glutamyl transferase, globulin, sodium, chloride, cobalt, iron, manganese, and selenium concentrations were significantly reduced. Affected camels were treated by stomach tube or surgery and were completely recovered, except for one camel with an esophageal fistula.

**Conclusion:**

A lack of trace elements could have a significant role in esophageal obstruction in dromedaries. Clinical, ultrasonographic, and hematobiochemical evaluations are useful for the accurate diagnosis, prognosis, and treatment of esophageal obstruction in camels.

## Introduction

Esophageal obstruction is a common and serious disorder in camels, contributing to indiscriminate feeding habits in these animals. The condition has significant consequences for animal health and economics; moreover, it is a common cause of esophageal surgery in camels [1, 2]. Esophageal obstruction commonly occurs at the cranial cervical esophagus, thoracic inlet, and cardia [3–6]. Improper husbandry hygiene is often cited as a principal cause of high esophageal obstruction rates [5]. Moreover, esophageal obstruction is related to nutritional deficiency and imbalanced nutrient intake, particularly micronutrients, including copper (Cu), iron (Fe), cobalt (Co), manganese (Mn), and selenium (Se). Common organic foreign bodies and materials, such as plastic bags, cloth, hairballs, and food often comprise the objects found in the esophagus [7–9].

A clinical diagnosis of esophageal obstruction can be inconclusive. However, advanced non-invasive diagnostic techniques, such as radiography, ultrasonography, and hematological and biochemical examinations can provide a definitive diagnosis [10]. In addition, exploratory surgery can be used to confirm the condition [11–13].

Several studies have described esophageal obstruction in camels [1, 6, 7, 14, 15]. Despite the prevalence and popularity of camels, little evidence of esophageal obstruction occurrence and diagnosis rates, and also assessment of trace element deficiency, have been recorded in the literature. Therefore, this study aimed to assess the effects of mineral deficiency on esophageal obstruction rates in dromedaries and to describe their clinical presentation and treatment outcomes.

## Materials and Methods

### Ethical approval

The study was approved by Committee for Animal Welfare and Ethics in accordance with the Laboratory Animal Control Guidelines of Qassim University (No. 367).

### Study period and location

The study was conducted from January 2019 to January 2020 on the cases admitted to the Veterinary Teaching Hospital, Faculty of Veterinary Medicine, Qassim University.

### Camels

Twenty-eight camels were allocated to two groups. Group 1 (control) consisted of 10 sound camels, whereas 18 camels with an esophageal obstruction history were allocated to Group 2. Animals ranged in age from 1 to 96 months (mean ± standard deviation [SD] = 84 ± 6 months), weight = 70–450 kg (mean ± SD = 280 ± 90 kg), and were of different breeds.

### Clinical examinations

At admission, camels were clinically and subjectively assessed for esophageal obstruction. Body temperature and vital signs were reported for all animals. Animal data, which included age, breed, sex, obstruction duration (time from esophageal obstruction and admission), surgical intervention, and foreign bodies, were assessed, compared, and statistically analyzed (Table-1).

**Table-1:** Clinical findings and surgical outcomes of esophageal obstruction in 18 dromedary camels.

Case No.	Breed	Age	Sex	Duration	Type of foreign body	Surgical interference	Outcome
1.	Wadeh	2 month	Male	4 days	Cloth	Esophagotomy	Recovered
2.	Wadeh	5 years	Male	5 days	Piece of carpet	Esophagotomy	Recovered
3.	Asfar	1.5 month	Male	3 days	Cloth	Esophagotomy	Recovered
4.	Asfar	4 years	Male	4 days	Feed material	Esophagotomy	Recovered
5.	Mejhem	3 years	Female	3 days	Feed material	Stomach tubing	Recovered
6.	Wadeh	2.5 month	Female	3 days	Cloth	Esophagotomy	Recovered
7.	Mejhem	8 years	Female	2 days	Cloth	Esophagotomy	Recovered
8.	Wadeh	4 month	Male	3 days	Cloth	Esophagotomy	Recovered
9.	Mejhem	6 month	Male	2 days	Clothes	Esophagotomy	Esophageal fistula
10.	Asfar	8 month	Female	3 days	Hair ball	Esophagotomy	Recovered
11.	Wadeh	1 month	Male	1 day	Plastic bag	Esophagotomy	Recovered
12.	Mejhem	7 years	Female	3 days	Feed material	Esophagotomy	Recovered
13.	Ashaal	2 month	Male	2 days	Plastic bag	Esophagotomy	Recovered
14.	Wadeh	9 month	Male	2 days	Plastic bag	Esophagotomy	Recovered
15.	Mejhem	10 years	Female	4 days	Feed material	Stomach tubing	Recovered
16.	Wadeh	10 years	Male	3 days	Cloth	Esophagotomy	Recovered
17.	Wadeh	5 years	Male	2 days	Cloth	Esophagotomy	Recovered
18.	Wadeh	3 month	Female	1 day	Cloth	Esophagotomy	Recovered

### Hematobiochemical examinations

Hematological examinations were performed by taking 5 mL venous blood from the jugular vein into ethylenediaminetetraacetic acid tubes using the VETSCAN HM5 (Abaxis Inc., Union City, CA, USA). Blood analyses included total and differential leukocyte counts, red blood cells, hematocrit, hemoglobin, mean corpuscular hemoglobin, mean corpuscular hemoglobin concentration, and mean corpuscular volume.

Biochemical examinations were also performed by taking 10 mL jugular vein blood, without anticoagulant addition. Blood samples were at 1500× *g* for 5 min to produce serum for biochemical analyses. Total protein, albumin (ALB), globulin (GLOB), glucose (GLU), blood urea nitrogen (BUN), creatinine (CR), creatine kinase (CK), aspartate aminotransferase (AST), γ-glutamyl transferase (GGT), alkaline phosphatase (ALP), alanine aminotransferase (ALT), calcium, phosphorus, magnesium, sodium (Na), and potassium were measured using an automated biochemical analyzer (VETSCAN VS2, Abaxis Inc., Union City). Furthermore, using inductively coupled plasma-mass spectrophotometry (ICP-MS, model no. 7800; Agilent, Santa Clara, CA, USA), zinc, Cu, Fe, Co, Mn, chloride (Cl), and Se concentrations were determined.

### Radiographic examinations

Dorsoventral and lateromedial standard radiographs of affected camels were obtained using a Minx ray HF 100/30 generator (Toshiba, Tokyo, Japan) according to El-Shafaey *et al*. [16]. Camels were lightly sedated using intravenous (IV) injection of 0.1 mg/kg xylazine HCl (2%). Radiographs were subjectively interpreted before surgical interventions.

### Ultrasonographic examinations

Ultrasonographic evaluations were performed on lightly sedated camels in lateral recumbency positions using 3.5–7.5 MHz linear transducers (Aloka, Tokyo, Japan) [17]. The ventral aspect of the neck was prepared for the ultrasonographic examination of esophageal obstructions.

### Surgical treatments

**Figure-1:**
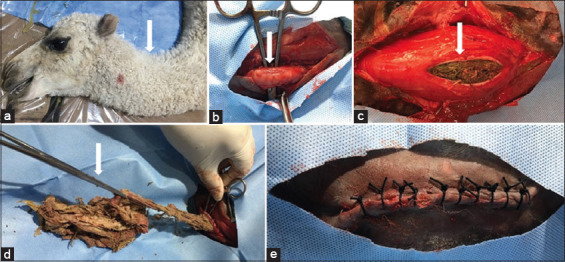
(a) The camel was placed in the right lateral recumbency. (b) Surgical exposure of the esophagus during esophagotomy. (c) Incision of the esophageal wall. (d) Surgical extraction of the obstructive foreign body using sponge forceps. (e) Skin was then sutured by silk No. 2 (United medical industries Co. Ltd. Riyadh, Kingdom of Saudi Arabia) and simple interrupted stitches.

Based on the aforementioned diagnostic investigations, esophagotomy was performed to remove esophageal foreign bodies from affected camels after medicinal treatment and/or stomach tubing strategies had failed [1]. Esophagotomy was performed under sedation using an IV injection of 3 mg/kg xylazine HCl and the linear infiltration of 10 mL lidocaine HCl (2%) at the incision site. The camel was placed in a right lateral recumbency position (Figure-1a), with the operation site thoroughly and aseptically prepared. A longitudinal skin incision (8–10 cm) was applied to the ventrolateral aspect of the neck, caudal to the foreign object. Neck muscles were bluntly dissected to approach the esophagus (Figure-1b), which was bluntly separated and elevated from underlying tissue using curved artery forceps before opening its wall. A long sponge forceps was advanced through the esophageal wound to grab and extract the foreign body (Figures-1c and d). The esophageal wall was cleaned and closed using one row of lambert suture pattern stitches with polydiaxanone (PDS) no. 00 (Ethicon, UK). Cervical muscles were routinely sutured in a simple continuous pattern using PDS no. 1, while the skin was sutured with simple interrupted stitches using PDS no. 1 (Ethicon) (Figure-1e). Following surgery, a pre-operative antibiotic was continued for 10 days and anti-inflammatories continued for 5 successive days, in addition to an intramuscular (IM) injection of 10 mL vitamin AD3E (ADVIT-DE, Morvel Laboratories P. Ltd., India). The camel was confined to stall rest for 4 weeks with daily monitoring of the healing process. Camels were postoperatively discharged from the clinic at approximately 4 weeks. To evaluate long-term health conditions, a telephone survey with owners was conducted to inquire about the surgical site and general surgery outcomes. A postoperative IM injection of 1 mg/kg cefquinome sulfate (Cobactan^®^ 2.5%, MSD Animal Health, Rahway, NJ, USA) was administered for 5 successive days. Furthermore, IV injections of 0.5 mg/kg meloxicam (Rheumocam; Chanelle Pharma, Galway, Ireland) were administered daily for three doses preoperatively. Moreover, an IM injection of 50,000 IU/kg AD3E (Kepro, Maagdenburgstraat, Netherlands) was also administered. Each operated camel was housed in a stall for daily dressing and monitoring the healing process for about 4 weeks postoperatively. A phone contact with the owners was performed to evaluate the functional outcome of the surgery to assess the long-term health condition results of the treated animal.

### Statistical analysis

Normal data distribution was assessed using the homologous disapproved theory. Unpaired t-tests were used to compare mean variable values between affected and control animals. Data were considered significant at p < 0.05.

## Results

### Clinical findings

Eighteen dromedary camels (Group 2) were clinically diagnosed with esophageal obstruction. Of these animals, the obstruction rate was higher in Wadeh (n = 9; 50%) camels when compared with Mejhem (n = 5; 27.8%), Asfar (n = 3; 16.7%), and Ashaal camels (n = 1; 5.5%). Furthermore, obstruction rates were higher in calves when compared with older camels (10 vs. 8; 55.5%), and also in males (n = 11; 61.1%) when compared with females (n = 7; 38.9%). Affected camels showed a sudden onset of dysphagia, salivation, and food regurgitation directly after suckling or eating. The owners observed these signs 1–5 days before hospital admission. Stomach tubing pushed the obstructive mass toward the rumen in two camels and failed in 18 camels, which suggested complete esophageal obstruction at the thoracic inlet (Table-1).

### Hematobiochemical findings

Hematological analyses showed significant increases (p < 0.05) in neutrophils, lymphocytes, and monocytes. However, compared to controls, significant decreases in total white blood counts were observed in camels with esophageal obstruction (Table-2). Biochemical findings showed significant increases in AST, ALT, ALP, CK, GLU, ALB, CR, and BUN concentrations. However, CR and BUN concentrations were significantly higher in affected camels when compared with controls. However, significantly decreased GGT, GLOB, Na, Cl, Co, Fe, Mn, and Se concentrations were recorded in affected camels compared to controls (Table-3).

**Table-2:** Mean ± SE, mean difference, 95% confidence interval of difference, and p-value of hematological examination of the control camels and 18 dromedary camels with esophageal obstruction.

Parameter	Mean ± SE		Mean difference	95% Confidence interval of difference		p-value
	
	Control	Diseased		Lower	Upper	
Red blood cells (10^12^/L)	11.1 ± 1.3	11.69 ± 0.46	11.69	10.65	12.72	0.000
Hemoglobin (g/dL)	16.2 ± 2.4	18.80 ± 1.23	18.80	16.06	21.55	0.000
Hematocrit (packed-cell volume) (%)	28.7 ± 2.6	28.32 ± 0.80	28.32	26.53	30.12	0.000
Mean corpuscular volume (fL)	25.3 ± 1.4	24.10 ± 0.54	24.10	22.89	25.32	0.000
Mean corpuscular hemoglobin (pg)	14.9 ± 2.5	16.10 ± 0.43	16.10	15.12	17.07	0.000
Mean corpuscular hemoglobin concentration (g/dL)	57.8 ± 9.1	66.65 ± 2.91	66.65	60.14	73.15	0.000
red cell distribution widths (%)	25.2 ± 0.3	29.63 ± 1.82	29.63	25.55	33.71	0.000
White blood cells (10^9^/L)	170.000 ± 2.800	78.45 ± 22.03	78.45	29.35	127.56	0.005
Lymphocytes	5.800 ± 2.300	2112.07 ± 881.58	2112.07	147.78	4076.36	0.038
Monocytes	0.09 ± 0.02	0.2182 ± 0.048	0.2182	0.10	0.32	0.001
Eosinophils	2.05 ± 0.57	1.05 ± 0.24	1.05 ± 0.24	0.51	1.60	0.001
Neutrophils	9.900 ± 3.100	3613.15 ± 1502.84	3613.15 ± 1502.84	264.60	6961.70	0.037

p < 0.05 was considered significant, SE=Standard error

**Table-3:** Mean ± SE, mean difference, 95% confidence interval of difference, and p-value of biochemical examination of control camels and 18 dromedary camels with esophageal obstruction.

Parameter	Mean ± SE		Mean difference	95% Confidence interval of the difference		p-value
	
	Control	Diseased		Lower	Upper	
Sodium (mEq/L)	156.5 ± 2.9	144.45 ± 6.38	144.45	130.22	158.68	0.000
Potassium (mEq/L)	3.8 ± 0.3	4.53 ± 0.50	4.53	3.41	5.65	0.000
Calcium (mg/dL)	8.4 ± 0.7	8.98 ± 0.41	8.98	8.06	9.90	0.000
Phosphorus (mg/dL)	2.7 ± 0.4	5.85 ± 0.96	5.85	3.69	8.01	0.000
Chloride (mEq/L)	5089 ± 168.82	4812.54 ± 145.13	4812.54	4489.16	5135.92	0.000
Zinc (ppm)	11.91 ± 83	11.33 ± 0.33	11.33	10.58	12.07	0.000
Copper (ppm)	11.09 ± 0.57	11.53 ± 0.87	11.53	9.57	13.48	0.000
Iron (ppm)	142.1 ± 6.83	114.01 ± 7.32	114.01	97.69	130.33	0.000
Cobalt (ppm)	300 ± 17.13	265.32 ± 13.19	265.32	235.92	294.72	0.000
Manganese (ppm)	3.817 ± 0.08	3.67 ± 0.06	3.67	3.53	3.82	0.000
Selenium (ppm)	119.91 ± 0.63	101.28 ± 4.50	101.28	91.24	111.31	0.000
Magnesium (mg/dL)	0.25 ± 0.03	1.56 ± 0.36	1.56	0.75	2.37	0.002
Blood urea nitrogen (mg/dL)	18 ± 10.1	31.09 ± 6.51	31.09	16.56	45.61	0.001
Creatinine (mg/dL)	0.95 ± 1.30	2.34 ± 0.64	2.34	0.90	3.79	0.005
Aspartate aminotransferase (IU/L)	68 ± 44	94.36 ± 15.75	94.36	59.26	129.46	0.000
Alanine aminotransferase (IU/L)	17 ± 0.70	20.63 ± 2.96	20.63	14.02	27.24	0.000
alkaline phosphatase	7 ± 3	87.54 ± 26.74	87.54	27.96	147.12	0.008
Creatine kinase (IU/L)	138 ± 22	215.72 ± 32.98	215.72	142.22	289.22	0.000
Gamma-glutamyl transferase (UL)	13 ± 5.0	8.81 ± 1.18	8.81	6.18	11.45	0.000
Total bilirubin mg/DL	0.40 ± 0.07	0.40 ± 0.009	0.40	0.38	0.42	0.000
Albumin (g/dL)	4.3 ± 0.4	5.04 ± 0.36	5.04	4.22	5.86	0.000
Total proteins (g/dL)	7.4 ± 0.4	7.16 ± 0.50	7.16	6.04	8.28	0.000
Globulin (g/dL)	3.8 ± 0.5	2.94 ± 0.31	2.94	2.23	3.65	0.000
Glucose mg/dL	62 ± 19.3	165.90 ± 31.55	165.90	95.60	236.21	0.000

SE=Standard error, p *<* 0.05 were considered significant

### Radiographic findings

**Figure-2:**
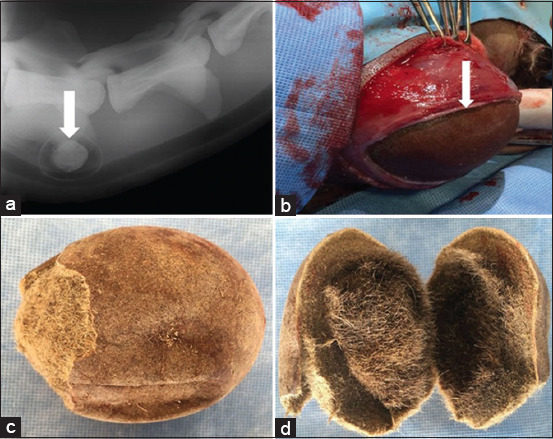
(a) Lateral radiographic view revealed a radiopaque hairball lodged within the lumen of the esophagus in a camel calf. (b) Surgical removal of a hairball in the same case in A. (c and d) The surgically removed hairball in the same case in A.

**Figure-3:**
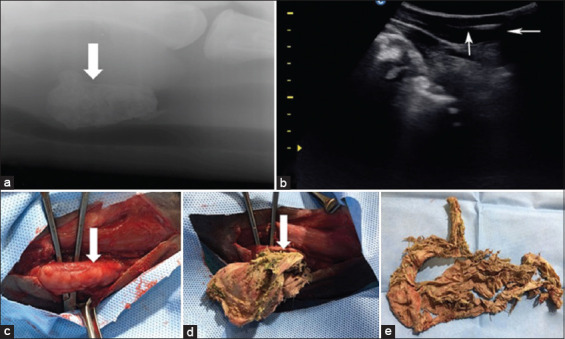
(a) Lateral radiograph, showing the presence of a soft tissue mass density (a piece of cloth) within the lumen of the esophagus in a camel. (b) Ultrasonographic examination of the esophagus shows a piece of cloth (arrow), diagnosed as an echogenic line within the lumen of the esophagus in the same case. (c) Surgical exposure of the esophagus during esophagotomy in the same case. (d) Surgical extraction of a piece of cloth in the same case. (e) The extracted piece of cloth in the same case; note the foodstuff with the piece of cloth.

**Figure-4:**
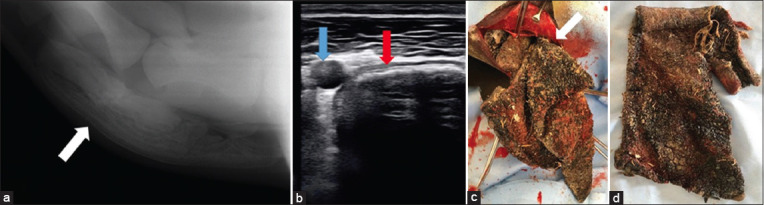
(a) Lateral radiograph, showing the presence of an irregular soft tissue mass density (a piece of carpet) lodged within the lumen of the esophagus in a 5-year-old male Wadeh camel with a gaseous radiolucency within the dilated esophagus cranial to the foreign body. (b) Ultrasonographic examination of the esophagus showing a piece of carpet (blue arrow), diagnosed as an echogenic mass within the variable amount of anechoic accumulated fluids in the esophagus (red arrow) in the same case. (c) Surgical exposure of the esophagus and surgical extraction of the piece of carpet during esophagotomy in the same case. (d) The extracted piece of carpet in the same case; note the foodstuff with the piece of carpet.

Esophagus radiography of affected camels showed more or less obstructive foreign bodies in the esophageal lumen. In some cases, foreign bodies appeared as irregular soft tissue masses with a gaseous radiolucency in the dilated esophagus cranial to the foreign body. Critically, tracheal radiolucency acted as a negative contrast background for most foreign bodies, either with high or low radiodensity (Figures-2a, 3a, and 4a). A positive diagnosis was confirmed by esophagotomy.

### Ultrasonographic findings

Ultrasonographic examinations identified increased reductions in esophagus diameters in affected animals (n = 18), accompanied by different anechoic fluid volumes in the lumen. Ultrasonographic features of obstructive foreign bodies differed according to their nature; most were hyperechoic materials. Cloth pieces (n = 9) were the most common objects in affected camels and were precisely ultrasonographically diagnosed as echogenic masses in the esophageal lumen (Figure-3b). A piece of carpet and a hairball were the least common bodies; they were diagnosed as echogenic masses in the esophageal lumen surrounded by different anechoic fluid volumes (Figure-4b). In addition, plastic bags in camels were identified as irregular hyperechoic masses with distal shadowing. Other esophageal contents included ingesta and fluids, which appeared as hypoechoic and anechoic masses, respectively.

### Treatment outcomes

Surgically extracted foreign bodies included cloth (n = 9) (Figures-3c–e), food materials (n = 4), plastic bags (n = 3), a hairball (n = 1) (Figures-2b–d), and a piece of carpet (n = 1) (Figures-4c and d). Cloths were the most common foreign materials, with the least common being a hairball. No foreign bodies were metallic. A 6-month postoperative follow-up showed that treated camels were completely recovered, except for one which had an esophageal fistula and was routinely treated until completely healed (Table-1).

## Discussion

In this study, camels (n = 16) undergoing surgery had obstructive foreign bodies in the esophagus. This was possibly related to nutritional deficiencies and mineral imbalances, especially trace elements and poor farming and hygiene in grazing areas. These findings conformed to the previous studies [5, 18, 19].

The relationship between camel breed and esophageal obstruction was clear in our study; the highest obstruction rates were observed in Wadeh camels when compared to other breeds (9 vs. 9). This observation was primarily due to the prominence of Wadeh camels in Saudi Arabia, and also to its reproductive and productive status [20]. In our study, camels under 1 year old exhibited more esophageal obstruction rates (55%, n = 10) when compared with older camels. These findings concurred with the previous studies [1, 11, 21] and may be attributed to the uncontrolled rearing environmental pollution facilitating their availability to camels’ especially young camel calves playing and consequently ingesting them.

We showed that gender exerted effects on esophageal obstruction rates, especially in male camels, when compared with females (11 vs. 7; 61.1%–38.9%). This was potentially attributed to associations between esophageal obstruction and male camel grazing in polluted desert areas containing plastic products, while females were often restricted indoors, especially during pregnancy periods. This finding coincided with a study by Ahmed [1].

Different foreign bodies were discovered in the esophagus of affected camels during esophagotomy. Cloths were the most common bodies, followed by plastic bags, a hairball, and carpet pieces. This may have been due to nutritional deficiency and mineral imbalance in camel rations, such as Na and Cl deficiencies and also trace element deficiencies, such as Cu, Fe, Co, Mn, and Se. These results agreed with previous studies [5, 22].

Hematological changes in camels with esophageal obstructions were comparable with controls. A significant increase in neutrophils, lymphocytes, and monocytes in affected camels was observed and was possibly related to inflammatory responses to infections associated with foreign bodies. Alternatively, significant reductions in total white blood counts were possibly attributed to reduced cell immunity associated with esophageal obstruction-mediated stress. These observations are in accordance with the previous studies [22–24].

We observed significant reductions in Na and Cl serum levels in affected camels, which possibly contributed to dysphagia and fluid loss due to excessive salivation. Furthermore, significant decreases in trace elements, including Co, Fe, Mn, and Se, were possibly related to improper rations, which may have caused animals to ingest foreign materials. These findings were similar to previous studies [5, 18]. Liver function tests revealed significant increases in AST, AP, ALT, and C concentrations, which indicated health deterioration in affected camels. Similar results were reported in previous studies [22, 23, 25].

GLU levels in affected camels were significantly higher when compared with controls, which is consistent with a study conducted by Zahra *et al*. [26] but in contrast with Ghanem [22]. Hyperglycemia in esophageal obstruction cases may be due to fat depletion, which acts as a GLU source in camels during fasting and long-standing inflammatory conditions. Hemostasis processes were also different between affected and control animals; GLOB concentrations were significantly decreased in affected camels, possibly due to infection and inflammatory responses related to a traumatic injury from esophageal foreign bodies. Creatinine and BUN levels in affected camels were significantly higher when compared with controls. This might have been due to renal insufficiency related to dehydration or nephritis due to septicemia. These findings are similar to the previous studies [22, 23].

Different esophageal disorders in camels can be precisely diagnosed using radiography. In this study, most affected camels were easily diagnosed using survey radiography. Tracheal radiolucency provided a negative contrast background for most foreign bodies, with either high or low radiodensity. Similar findings were identified elsewhere [1, 3, 4, 27, 28]. An ultrasonographic examination is a non-invasive tool for the accurate diagnosis and prognosis of esophageal obstructive foreign bodies [29–33]. Based on ultrasonographic examinations in affected camels, most foreign bodies in the esophageal lumen were hyperechoic in different anechoic fluid volumes. These ultrasonographic findings are similar to previous studies [30, 31].

Esophagotomy is the gold standard when confirming an esophageal foreign body diagnosis and is considered the treatment of choice for esophageal obstruction in camels. In 16 camels with esophageal obstruction who underwent surgery, all were completely recovered and regained normal functions afterward. However, one camel was complicated with an esophageal fistula but was retreated successfully. These findings agreed with a study by Ahmed [1].

## Conclusion

Trace element deficiency could play a significant role in developing esophageal obstruction in dromedaries. Clinical, ultrasonographic, and hematobiochemical evaluations are useful for accurate diagnosis, prognosis, and treatment of esophageal obstruction in camels.

## Authors’ Contributions

MS and EE: Conceptualization and data curation and writing - review and editing of the manuscript. MS, SE, SA, FA, and EE: Formal analysis, and investigation. All authors have read, reviewed, and approved the final manuscript.
